# Surfactant incorporated preyssler polyoxoanion: a transition metal substituted Mo-PHP complex and its applications

**DOI:** 10.55730/1300-0527.3543

**Published:** 2023-01-06

**Authors:** Bharath SAMANNAN, Jothi SELVAM, Yi-Li LIN, Praveen PETER, Jeyabalan THAVASIKANI

**Affiliations:** 1 Department of Chemistry, Sacred Heart College (Autonomous), Tamil Nadu, India; 2 Department of Safety, Health and Environmental Engineering, National Kaohsiung University of Science and Technology, Kaohsiung, Taiwan

**Keywords:** Preyssler polyoxoanion, cationic surfactant, transition metal (Mo), antimicrobial resistance, dielectric studies

## Abstract

The complex was prepared with preyssler polyoxoanion and transition metal (Mo), a cationic surfactant as a connector. It has tuneable physical and chemical potential which has been exploited to study novel properties. A new technique of shock wave impulses is also used on the Mo-PHP complex. Extensive use of cationic surfactants could impact accumulation in the environment set off the surfacing of bacterial resistance. Due to the electrostatic binding to bacterial surface, the hydrophobic parts of cationic surfactants tend to penetrate bacterial cell walls and may cause membrane lysis and bacteria death. The surfactant-supported and direct release of metal ions from P_5_W_29_Mo against bacterial resistance has been explained schematically. The dielectric study helps to understand the dissociation of cations that generate polarons and the hopping mechanism with neighbouring vacant atomic sites. Structural analysis confirms the formation of cationic surfactant incorporated polyoxoanion (Mo-PHP). A hexagonal shape-like structure for the PHP complex has been observed. The Mo-incorporated PHP complex was characterized using UV-visible (UV), Fourier Transform-infrared (IR), Raman spectra, scanning electron microscope (SEM), energy dispersive spectroscopy (EDS), and X-ray diffraction (XRD) techniques.

## 1. Introduction

Polyoxometalate is an anion of polynuclear metal oxide with large structured varieties and unique chemical and physical properties [1]. The POMs can self-assemble using weak interaction; therefore, the coordination of metal ions forms a new structure [2]. The POM category is made up of polyhedra clusters of transition metal anions that have influenced researchers because of their source, topology, versatile, controllable shape and size, higher electronegative or oxo-enriched surfaces, and so on [3, 4]. It also has various applications in different fields such as catalysis, biology, and medicine [5–8]. The cationic charge and hydrophobic tail of surfactants are crucial components of their antimicrobial activity [9]. POMs show a strong affinity for cationic molecules, which is an appropriate anion [10–12]. The CTAB cationic surfactant combined with POM would be a preferable material for the preparation of organic-inorganic hybrid polyoxoanion for biological applications [13].

The preyssler polyoxoanion has PW_6_ units with two groups of three corner-sharing WO_6_ octahedra [14]. Generally, the polyhedra structures are the most potential skeletons of the polyoxometalates (POM). The preyssler polyoxoanion has some key advantages; for example, the internal cavity allows metal cations of suitable size and has a highly oxygen-rich surface. The POMs are thermally stable and also stable in the pH range of 1–10 [15, 16]. The PHP complex with oppositely charged surfactants [17] and its bacterial challenges are explained in this paper [2, 18]. The preyssler polyoxoanion bonding function at the HAS process helps to understand the surfactant interaction [19]. Bacteria have shown resistance to almost all toxic metal ions of environmental concern.

In this work, the impacts of surfactant-incorporated Mo-incorporated PHP and shock wave impulses complex have been discussed. This article focuses on bacterial resistance of PHP as pathogenic bacteria pose a health risk and resistant bacteria are globally important [20]. The synthesized complex has been analysed using various spectral techniques like FT-IR, Raman, and UV-vis. The effect of shock wave parameters such as the number of impacts, and ambient temperature of the complex was discussed and the possible bacterial resistance mechanism was proposed in this paper. The preyssler polyoxoanion complex as (PHP-1) and shock wave impulses as (PHP-2) were obtained successfully.

## 2. Materials and methods

### 2.1. Materials

All the chemicals were purchased from Merck and used without further purification. Sodium tungstate hydrates, sodium molybdate hydrate, cetrimonium bromide (CTAB), orthophosphoric acid were all purchased as analytical grades (~99%).

### 2.2. Preparation of Mo-incorporated preyssler polyoxoanion (PHP-1)

The synthetic route followed for the preparation of surfactant-incorporated Mo-doped preyssler polyoxoanion (K_14_ [NaP_5_W_29_MoO_110_]) was adopted with slight modification in the literature method [5, 15]. About 56 g of Na_2_WO_4_.2H_2_O (0.169 mmol) and 2 g of Na_2_MoO_4_.2H_2_O (0.008 mmol) were dissolved in 70 mL of dist. H_2_O and refluxed at 60 °C for few min, then the solution was brought down to room temperature (RT). Meanwhile, 30 mL of orthophosphoric acid was added drop-wise to the reflux under constant stirring. Then, the yellow solution was potted into a stainless Teflon autoclave bottle at 120 °C for the next 24 h, where the colour changed from yellow to dark green in the potted-off autoclave (i.e. RT). To this solution, 10 g of KCl is dissolved in 30 mL of dist. H_2_O, while refluxed under constant stirring and maintained for 30 min. A few drops of CTAB were added to this solution with continuous stirring for another 10–15 min. To collect the greenish solid, the solution was then heated up to dryness. After that, those greenish solids were exposed to the shock waves impulses [21] of 20 impacts for the PHP complex (hereafter named PHP-2) and analysed using the spectral techniques for further confirmation.

### 2.3. Loading of shock waves

A primitively designed desk surface with semiautomatic shock tube was used. This tube has generated shock waves using a shock wave of Mach number 2.2 for the prepared complex, whereas it ranges from 1 to 4.5 Mach number to make shock wave impulses. The tube consists of five variants: input reservoir, drive, sensors, driver, and diaphragm sections, respectively. To generate the supply, the drive and the pressure are used as input at ambient or RT. The Diaphragm helps to link the driver and driven parts together. Meanwhile, the pressure in the driver section is amplified via a compressor and at critical level, its diaphragm ruptures, and the waves generated travel into the driven and the sensor parts of the tube. Then, the as-synthesized complexes are exposed to 20 impacts with a 2.2 Mach number. The pressure maintained during the experiment was 2.0 MPa and it was acquired using a GSM carbonless paper diaphragm [22].

### 2.4. Antimicrobial resistance

By means of zone of inhibition, the bacterial resistance (AMR) has been observed. The common pathogenic bacteria such as *Staphylococcus aureus (S. aureus;* gram-positive*)* and *Escherichia coli (E. coli;* gram-negative*)* are used for the antimicrobial resistance. The material was placed over the drug ciprofloxacin disk to examine the antimicrobial resistance through ionized and metalized disk diffusion method. In short, the bacteria were cultured overnight in a flask-accommodated liquid Luria-Bertani (LB, 25 g/L) within incubator shaker at 37 °C. At the same time, 25 g/L fresh LB agar with the curing agent (agar, 15 g/L) was poured in a petridish, while solidified at ambient temperature (RT). Afterwards, the cultured *S. aureus* and *E. coli* mixture (200 μL) was extended evenly on the solidified LB agar plates. Subsequently, the PHP complex was sterilized under ultraviolet radiation for 0.5 h, while placed on the surface of the LB agar in the petri dish and let the bacteria grow overnight at room temperature. Meanwhile, the zone of inhibition was observed around the disk.

## 3. Results and discussion

### 3.1. IR

**Figure 1:**
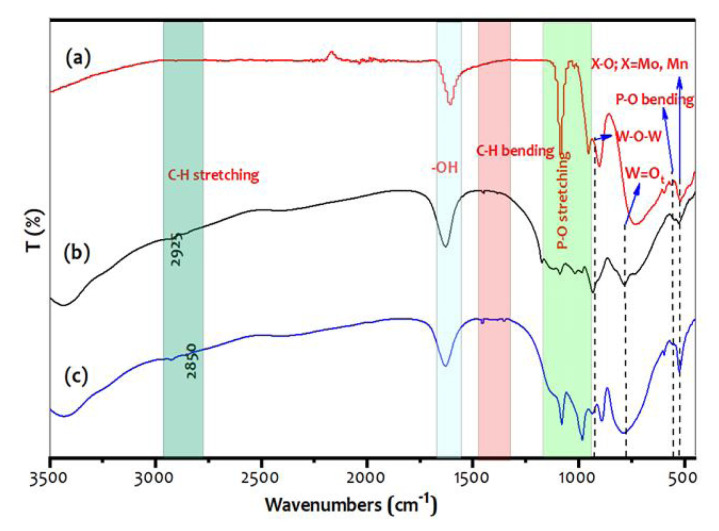
Fourier transforms infrared spectra of (a) Pure PHP, (b) PHP-2, and (c) PHP-1.

The IR spectra of the PHP complex are recorded at room temperature using IR, Spectrum 100, and Perkin Elmer. The functional groups were observed to have wave numbers ranging from 3500 to 500 cm^−1^ with KBr in mass 1:100 (sample: KBr). The IR spectral data of the preyssler polyoxoanions (PHP), PHP-1, and PHP-2 complexes recorded are shown in Figure 1. According to the literature, the vibrational peaks corresponding to the preyssler structure have some sharp/broad peaks which were also noticed [14, 23]. The finger-print region of the PHP complexes was as follows; the peaks at 1164–1115, 1087–1179, 1021–1016 cm^−1^ were assigned to the P-O stretching vibrations. The stretching frequencies of W-O-W are noticed at 977–948 cm^−1^ and the peak at 787–736 cm^−1^ is due to the presence of stretching W=O_t_ (terminal bridging oxygen) vibrations. One at 560–584 cm^−1^ indicates the P-O bending vibration. The peak at 526–535 cm^−1^ is attributed to metal complex X-O (X=Mo), whereas the small shoulder indicate the formation of the transition metal complex. The surfactant CTAB exhibits the peak at 1060–1020 cm^−1^ with a small hump, corresponds to hydroxyl group [24, 25]. The peak at 1452–1449 and 1348–1352 cm^−1^ corresponds to the bending frequency of the symmetric and asymmetric –CH_3_ group [26, 27]. Meanwhile, a broad peak at 3425–3431 cm^−1^ indicates the presence of a terminal hydroxyl group. The transition metal incorporated preyssler heteropolyacid PHP-1, and PHP-2 complex hold the core structural vibration frequencies as pure PHP.

### 3.2. UV-visible spectroscopy

**Figure 2:**
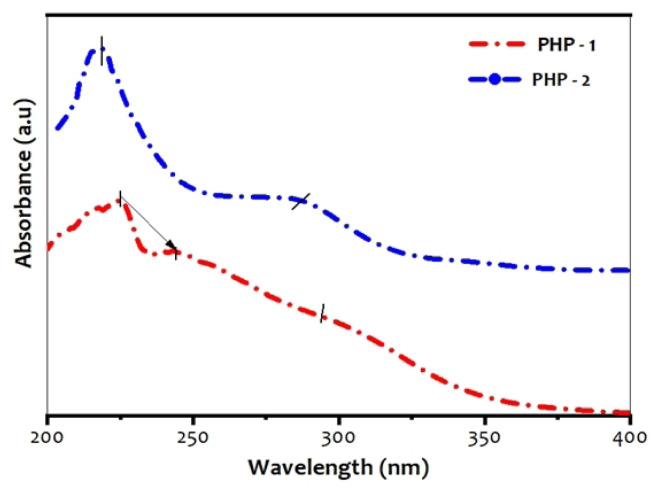
UV-visible spectra of (a) PHP-1 and (b) PHP-2.

UV-visible absorption spectra of Mo-incorporated PHP complex recorded is depicted in Figure 2. From the UV spectra, the two absorption bands corresponded to the PHP complex [13, 28]. The bands at 222 and 243 nm are attributed to the terminal bridging oxygen (W-Ot_1_) and (W-Ot_2_) bonds. One at 292 nm is assigned to interbridging oxygen (W-O_e_) vibrations of the PHP-1 complex. Furthermore, the bands at 218 nm and 280 nm were assigned to the stretching vibration of terminal-edge sharing (W-Ot) and interbridging oxygen (W-Oe) of the PHP-2 complex. As can be noticed, the absorption bands are blue-shifted (i.e. downwards) due to the formation of bonds and the particle grain size (218–222 and 280–292 nm for PHP-1, 2^n^). The absorption band (λ_max_) and band gap energy are tabulated in Table. From Equation 1, the band gaps for the PHP complexes are calculated.


(1) $${\text{E}}_{\text{g}}=\frac{1240}{\lambda \text{max}}(\text{eV}),$$

where E_g_ is the band gap energy and λ_max_ is the wavelength (nm).

The particle size in the material tends to change colour due to their absorbance of light [29]. For particles of small grain size, the band gap increases, whereas for the particles of larger grain size, it decreases. The PHP complex is synthesized as a light greenish solid and analysed using quantitative technique. The shock wave impulses PHP-2 complex absorbed higher region as compared to the PHP-1 complex. However, the absorbed bands confirm that there is no substantial change after the shock wave impacts.

### 3.3. X-ray diffraction (XRD)

**Figure 3:**
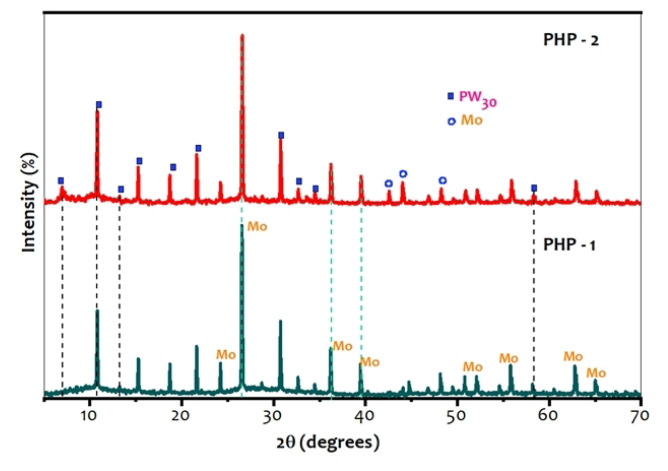
X-ray diffraction (XRD) spectral data of (a) PHP-1 and (b) PHP-2.

The XRD patterns were recorded for a PHP complex using Bruker D2 phaser in the range of 0–90°. XRD spectral data of Mo-incorporated PHP complex, namely PHP-1 and PHP-2, are given in Figure 3. From the literature, the pure preyssler polyoxoanion is known as [NaP_5_W_30_O_110_]^14−^, whereas diffraction peaks are experimental recorded as follows; 2θ = 6–10°, 15–22° and 24–30°, respectively [7, 15]. From the XRD spectral data observed, the peaks at 10.9°, 12.9°, 15.2°, 18.6°, 22.3°, 24.8°, and 30.5° correspond to PHP-1 of pure PHP [16]. The peaks at 2θ = 26.2°, 35.8°, 39.8°, 53.4°, and 63.9° are assigned to the indexed planes (222), (233), (115, 333), (444), and (147, 455) which confirm Mo in the complex [17]. The shock waves impulse PHP complex, 2θ = 42.3°, 43.5°, 47.8° with indexed planes (125), (044), and (116, 235), indicates the presence of the (Mo) in PHP-1 complex. This technique is used to identify the grain size of the material and unit cell dimensions of the hybrid material. The peak corresponding to the transition metal (Mo) is given in Figures 3a and 3b.

### 3.4. Scanning electron microscope/elemental compositions

**Figures 4:**
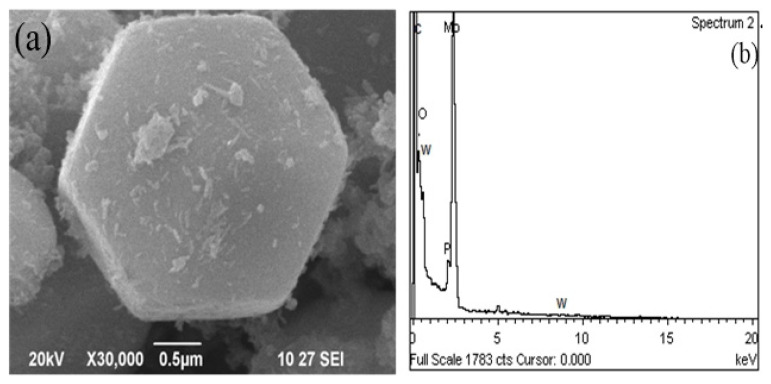
(a) SEM image and (b) elemental analysis of PHP complex (PHP-1).

Morphological studies of synthesized PHP complex are carried out by scanning electron microscope (SEM; FEI Quanta 200, USA), which is operated at 20 kV accelerating voltage with a resolution of 1–10 μm. All the samples were recorded using FEI Quanta 200, USA after sputtering a thin layer of Au on the synthesized samples to make the surface conductive for the elemental analysis of the prepared complexes [15]. The morphological microstructure of the Mo-incorporated PHP complex is noticed using SEM monitored is shown in Figure 4. These figures confirm the hexagonal shape structured material lies in the range of 1–1.3 μm. The SEM images evidently confirm that the morphology of the Mo-incorporated PHP complex exhibits six-sided polygons. The elemental composition of the PHP complex reveals the presence of corresponding elements observed through EDS analyses (wt. %): carbon (C), 12.18%; tungsten (W), 50.62 %; molybdenum (Mo), 3.82%; oxygen (O), 28.10%, and phosphorous (P), 5.18 %, respectively.

### 3.5. Raman spectroscopy

**Figure 5:**
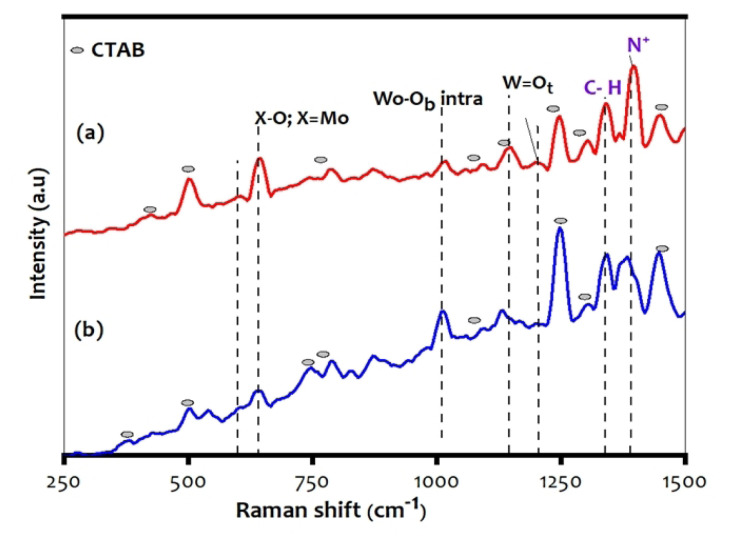
Raman spectra of PHP complexes (a) PHP-1 and (b) PHP-2.

Raman spectra of the Mo-incorporated PHP complex have spectral bands which are given in Figure 5. As per the literature, the absorption bands of preyssler polyoxoanion have been retained both in the pure and the hybrid complexes. The PHP bands at 970–998 and 662–669 cm^−1^ are attributed to the bridging oxygen (Wo-O_b_) and the stretching frequency of X-O; X = Mo bonds. The vibrations of tungsten terminal-oxygen have been observed with low intensity, indicating that vibration stretching is not pure. The surfactant-incorporated PHP complex is characteristics at 499–502, 791–795, 1070–1075, 1127–1130, 1295–1301, 1480–1485 cm^−1^ [7, 30]. Besides, the small and intense broad band at 1343–1345 and 1450–1452 cm^−1^ indicates the presence of the stretching frequencies of C-H and N-H (or N^+^ ions) spotted in the Raman spectral.

### 3.6. Antimicrobial resistance (AMR)

**Figure 6:**
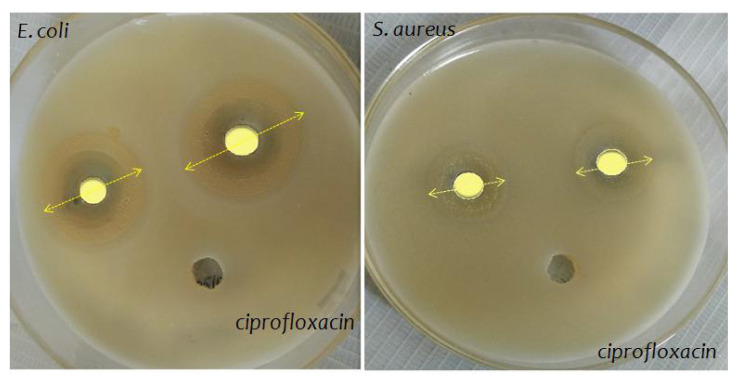
Antimicrobial resistance of the complex of (a) and (b) *E. coli* and *S. aureus* for PHP-1 (left side of each plate) and PHP-2 (right side of each plate).

**Figure 7:**
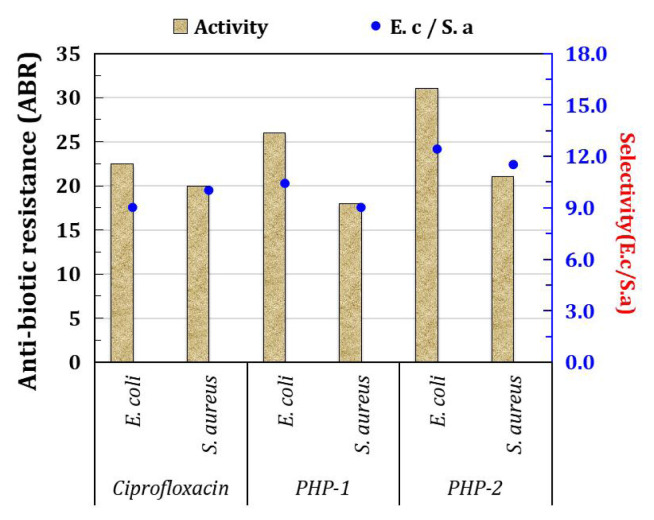
Bar chart of the PHP complex.

**Figure 8:**
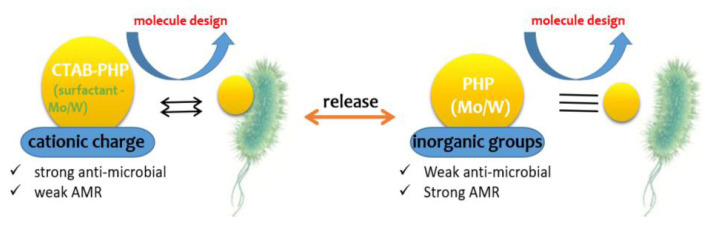
Possible mechanism of bacterial resistance of PHP complex.

The antimicrobial resistance (AMR) of the Mo-incorporated PHP complex noted in the presence of pathogenic bacteria like gram-positive (*S. aureus*) and gram-negative (*E. coli*) is presented in Figures 6a–6d). The bar chart of the antimicrobial resistance (i.e. zone of the inhibition (mm)) values is given in Figure 7. The PHP-1 complex against gram-positive (*S. aureus*) and gram-negative (*E. coli*) has been observed; inhibition zones of around ± 26 mm and ± 18 mm were measured via diameter. Besides, the PHP-2 complex shows that the zone of inhibition is around ±31 mm and ±21 mm of pathogenic bacteria. Meanwhile, aggregation properties derive from the accumulation of the cationic surfactant molecules on the surface of the cell membrane [31]. The PHP-1 complex shows less resistance, which is due to the minor aggregation through dispersion (well) as compared to the PHP-2 complex. The PHP complex without surfactant shows zone of inhibition at a negligible level. The PHP complex with surfactant interaction is apparent and large [32]. As shown in Figure 7, the absence of inhibition in both gram-positive (*S. aureus*) and gram-negative (*E. coli*) of without surfactant is noted. According to the previous report, the Mo/W inorganic group protects the pathogenic bacteria at a certain level [33, 34]. Incorporating Mo/W ions system (Mo-PHP) is a promising plan for antibacterial resistance against pathogenic bacteria agents, whereas Mo-PHP without cationic surfactant clearly shows no zone of inhibition. The results suggest that the selectively rendered K^+^ ions at RT can be controlled by the accumulation of ions in aqueous solution [35]. Furthermore, shock wave impulses PHP after 20 impacts still exhibit good antibacterial ability towards both bacteria as shown in Figure 7. The enhanced bacterial resistance may be due to the presence of the surfactant in the complexes [36]. The mechanism of bacterial resistance of PHP complex schematic is shown in Figure 8.

### 3.7. Dielectric studies

**Figures 9:**
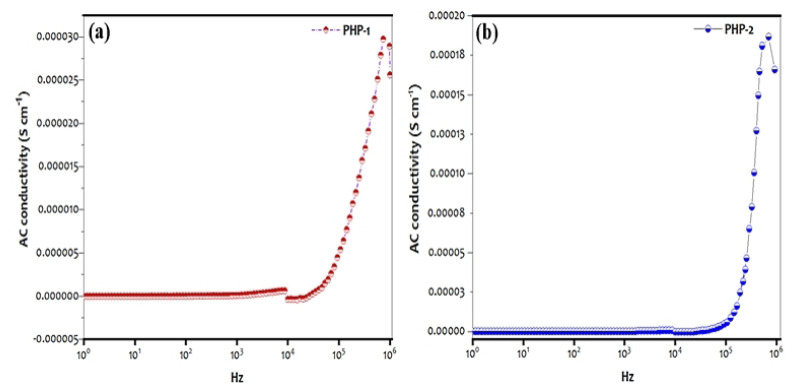
Conductivity studies of the complexes (a) PHP-1 and (b) PHP-2.

The conductivity of the PHP complex, which is measured at 313 K is given in Figure 9. As shown in Figure 9, the conductivity of the PHP-2 complex is 1.19 × 10^−4^ S cm^−1^ as compared to that of the PHP-1 (3.05 × 10^−5^ S cm^−1^) complex. The curve clearly shows that there are two different conductivity regions, which have been observed in both cases of the complex represented as region I and region II. The first stage of the plotted exhibit horizontal frequency-dependent line (100 Hz to 10^4^ Hz) may be due to the nonpolarization. As a result, a plateau-like frequency appears at the end of 10^3^–10^4^ Hz [37]. The relaxation hump in the region of 10^4^ Hz is due to their intermolecular charge transfer (CT), which has an assorted valence of the Mo-O or W-O stretching vibration. Meanwhile, the relaxation peaks above 10^5^ Hz causes dipolar relaxation of the distorted W-O-W lattice [37]. The binding site of CTAB counter-cations of N^+^ [38], NH^3+^ [13, 39] has attracted the anionic molecule.

**Figures 10:**
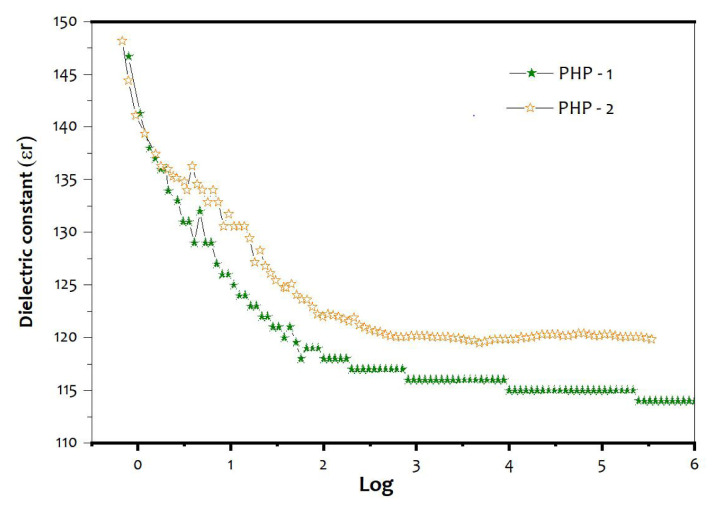
Dielectric constant (ɛr) of (a) PHP-1 and (b) PHP-2 complex.

**Figures 11:**
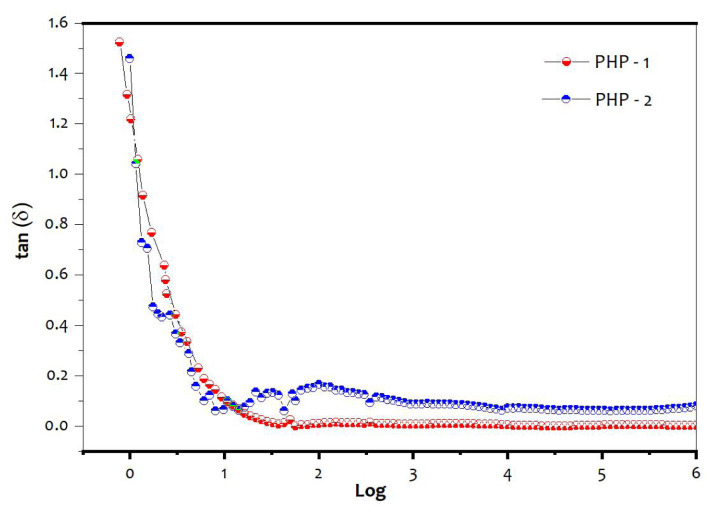
Dielectric loss (tan δ) of (a) PHP-1 and (b) PHP-2 complex.

**Figure 12:**
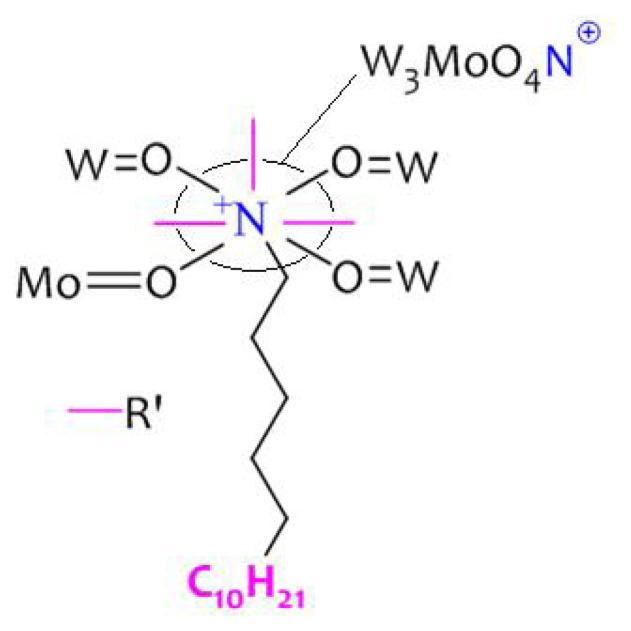
Schematic of the outer-ring of bulk proton sites in PHP complex.

Dielectric constant (ɛr) and dielectric loss (tan δ) of the PHP complexes are shown in Figures 10 and 11. The PHP-1 complex shows higher dielectric constant value of 148 (at 100 Hz) and dielectric loss of 1.57 (tan δ). The ɛr value of 146 (at 100 Hz) and dielectric loss of 1.48 (tan δ) of the PHP-2 are compared with those of the PHP-1 complex. The lattice water molecule and oxygen vacancies in the PHP complex cause distortion in the mobilizing charge carriers of oxygen atom [40, 41]. The above-mentioned process could have transfer charges where cationic surfactants possibly found in the PHP complex are present in Figure 12.

## 4. Conclusion

Surfactant supported Mo-incorporated PHP complexes were synthesized and analysed. This work explains the activity of antimicrobial resistance (AMR) and its possible charge transfer mechanisms. The optimal hydrophobicity may trigger the surfactant effectively to disrupt the bacterial lipid membrane. The concerned antimicrobial activity of cationic surfactant PHP and bacterial resistance with pure PHP compound have been discussed. In this study, the antibacterial activity with surfactant incorporated PHP that destroys or inhibits the growth of bacterial is noted. The Mo-incorporated PHP-2 complex exhibits higher bacterial activity as compared to the PHP-1 complex. Dielectric studies show that the PHP complex reveals higher degree of conductivity. Increasing polarizations affect the interaction of electron carrier with outer-ring proton sites. This complex may be used in other applications such as TFC membrane degradation and sewage water treatment.

## Figures and Tables

**Table:** UV-visible band and band gap energy observed for PHP-1 and PHP-2 complex.

Complexes	UV-vis absorption				
	λ_max_ (nm)			Band gap energy (eV)	
	Wo=O_t_	W-O_c_-W		Wo=O_t_	W-O_c_-W
PHP-1	222	292		5.5	4.2
PHP-2	218	280		5.6	4.4

## Abbreviations

CTAB: Cetrimonium bromide
SWH: shock waves assisted hydrothermal
PHP: surfactant supported Mo-incorporated preyssler polyoxoanion
POM: polyoxometalate
SEM: Scanning electron microscope
EDS: Energy dispersive spectroscopy
FT-IR: Fourier transform–infrared
UV: UV-Visible spectroscopy
Mo: Molybdenum
Na_2_WO_4_.2H_2_O: sodium tungstate hydrate
Na_2_MoO_4_.2H_2_O: sodium molybdate hydrate
KCl: potassium chloride
